# Scientific creativity and innovation ability and its determinants among medical postgraduate students in Fujian province of China: a cross sectional study

**DOI:** 10.1186/s12909-023-04408-9

**Published:** 2023-06-16

**Authors:** Fengqiong Liu, Shuming Qu, Yi Fan, Fa Chen, Baochang He

**Affiliations:** 1 Department of Epidemiology and Health Statistics, School of Public Health, Fujian Medical University, Fuzhou, China; 2 Department of Health Management, School of Health Management, Fujian Medical University, Fuzhou, China

**Keywords:** Scientific creativity, Medical education, Postgraduate, Cross-section

## Abstract

**Background:**

Graduate education is the main approach to training high-level innovative elites. With the expansion of the scale of graduate education in China, it has gradually emerged that the lack of innovation ability of graduate students is recognized as the primary problem in graduate education. How to comprehensively improve the quality of postgraduate teaching has become the core task of educational reform and development. However, data about the current cultivation and development of the innovative ability of graduate students in China is limited.

**Methods:**

A questionnaire survey was conducted among medical postgraduate students. Descriptive statistics and multiple regression analysis methods were used to analyze the data to describe the current innovation ability in advanced medical education and potential influence factors.

**Results:**

A total of 1241 medical students were surveyed, according to the results of questionnaire data analysis. The proportion of subjects who participated in the College Student’s Entrepreneurship and Innovation program or any other scientific research programs are fairly high which are 46.82% and 29.20% respectively. Most of the participants are observed with high levels of self-motivation and active learning and have good performance in creative thinking. However, only a small number of participants (16.6%) reported academic achievements such as publications. Most of the students are satisfied with the current scientific research environment and think that the current postgraduate training system is qualified for the cultivation of innovation ability, and expects the inclusion of course specialized in systemic medicine and medical informatics in the curricula. Multiple logistic regression results showed that among the factors studied, gender, medical specialties, and types of master’s degrees are associated with cognition & skills, academic performance, and creativity.

**Conclusions:**

It will be important to incorporate more techniques for creating and improving creativity in the curricula of the current postgraduate education, especially for courses such as systemic medicine and informatics. Guidance in earlier school life can stimulate creativity and an early introduction to scientific research work will facilitate innovative thinking and behavior. Scientific research programs such as the National Innovation and Entrepreneurship Training for the universities of PRC have been widely implemented in the undergraduate education system throughout the country. However, the training effectiveness of the current scientific research programs is worth improving.

## Background

As an important aspect of learning, creativity is an indicator of student development and a key learning outcome of the core business of education. In 2018, the OECD program in its publication “Learning Framework for 2030” mentioned that creativity is a necessary skill for learners, and recommend it as an educational focus [1]. To promote the social and economic development of society, we need talented people with a variety of twenty-first-century skills, cross-cultural communicative competence, creativity, innovation, and entrepreneurship. Twenty-first-century skills development is a target of many educational programs worldwide [2].

The importance of creativity in the medical sciences is to the extent that healthcare personnel often need creative solutions to interact with patients and clients and to make decisions and solve specific problems that arise in their careers and services [3, 4]. Medical students must master a huge amount of courses and topics in a limited time. They must acquire comprehensive knowledge of the subjects and good communication skills to take care of their patients. Therefore it is necessary to incorporate various techniques for creating and improving creativity in their curricula [5]. Therefore, universities should play an important role in the generation, growth, and development of creativity, and they are responsible for the identification of the current status of creativity in students, as well as its barriers and causes, and the results of such studies can be effective in planning to improve creativity and growth [6–8].

Since in each society, students as intellectual and creative human resources have a special position, it is necessary to identify the required conditions for educating qualified people, especially in the medical sciences and in medical students pursuing an advanced education degrees, who will be in charge of the health care of others after graduation. Therefore, this study aimed at evaluating creativity and its influencing factors among postgraduate students of medical universities in Fujian province, Southeast China.

Though several recognized tests have been widely used for the assessment of innovation capacity, they are mainly designed for the evaluation of generic skills and creative behavior, which could not directly reflect the innovation capacity and behavior of students of specific fields [9–11]. However, the evidence for domain specificity of creativity is found both in broadly defined cognitive domains, and in narrowly defined tasks or content domains, and the specificity of demands in each task was better identifiers than cognitive processes related to creativity [12]. So far there is no evaluation criteria and system established as to how to measure and evaluate the research and innovation ability of students with advanced education, not to mention tests or questionnaires specifically designed for medical students. Instead of generally used measurement approaches for creativity and innovation ability such as the Torrance Test of Creative Thinking (TTCT), Scientific-Creative Thinking Test, and the Persian version of the creativity questionnaire [13], we designed a questionnaire specifically focused on scientific research-centered ability in medical students, which concerns terms of creative thinking, cognition & skills in medical science, academic performance, attitude to the current training system. The results of the study will provide valuable insights into the students’ experiences, their perceptions of their training, and the current state of postgraduate education in China, and give a brief view of the current cultivation of innovation capabilities in medical students in China. The study also will shed light on the potential influence factors on innovation ability, and the factors associated with cognition and skills, academic performance, and creativity, which are important for informing the development of effective strategies to enhance innovation ability in medical postgraduate students.

## Methods

### Participants

In this cross-sectional study conducted in 2022, a total of 1241 postgraduate students from the Fujian Medical University, which is the largest medical university and medical training center for Fujian province in Southeast China, completed the questionnaire survey and was included in the study. A proportional random sampling method was used for this study. The researcher proportional selected students according to their professional background, which contained ten research fields including clinical medicine, public health, preventive medicine, nursing, preclinical medicine, pharmaceutical science, stomatology, medical imageology, clinical laboratory medicine, clinical anesthesia, and public health administration. The creativity questionnaire was anonymous self-administered to the participants whose participation in the study was voluntary. The proposal for this study was approved by the Institutional Review Board (IRB) of Fujian Medical University.

### Measures

The questionnaire consists of 46 items and it contains three parts. In part one, item 1–6 shows the characteristics of study subjects. In part two, items 7–38 measure three scales of scientific creativity and innovation ability, which consisted of creative thinking, cognition & skills in medical science, and academic performance, which were measured by items 7–13, 14–27, and 28–38 respectively. In part three, items 39–46 evaluated students’ cognition of innovation cultivation and attitudes to the current training system.

Scores were calculated separately for the three sections of creative thinking, cognition & skills in medical science, and academic performance. A higher score indicates a higher potential for creativity and innovation ability in medical science. Each item was scored on a 2-point scale ranging from 0 to 1 where 0 indicated low creativity, while 1 indicated high creativity.

### Reliability and validity test of Questionnaire

The reliability and validity of the QS were analyzed to ensure the accuracy and scientificity of the survey results. Reliability refers to the degree of internal consistency and external stability of the survey results. After the collected data were sorted out, the invalid QS were eliminated, as well as the questions with a factor load less than 0.5, and the questionnaire data are analyzed through the confirmatory analysis method. The results show that the overall internal consistency reliability of the scale is 0.80, and the α coefficient of the sub-dimension is between 0.65 and 0.79. In terms of structural validity, the correlation analysis method is used to test the structural validity of the scale, that is, the validity of the scale is estimated by the correlation between various factors in the scale and the correlation between each factor and the total score of the scale. The results show that the correlations between different dimensions and the total score range from 0.55 to 0.76, and the correlations between dimensions range from 0.36 to 0.60, which are in line with the statistical indicators of the scale.

### Data Analysis

All data analyses were carried out using the software R (version 3.1.1). Descriptive statistics were calculated to show the characteristics of the variables. The total score of scientific creativity and innovation ability was divided into low and high creativity groups according to the median score and then was used as the outcome variable. Multivariate logistic regression analysis was carried out by incorporating the variables that were significant below 0.05 in the Chi-square test to explore the potential factors related to the score of scientific creativity and innovation ability. The level of statistical significance was set at α = 0.05.

## Results

### Descriptive analysis

The statistical information of participants is shown in Table 1. 489 (39.4%) participants were male and 752(60.6%) were female. Most of the students (82.84%) are in the first academic year, 197 students (15.87%) were in the second and 16 students (1.29%) were in the third academic year. In terms of professional background, the proportion of participants from clinical medicine, public health and preventive medicine, nursing, preclinical medicine, pharmaceutical science, stomatology, medical imageology, clinical laboratory medicine, clinical anesthesia, and public health administration was 67.53%,3.71%,2.9%,2.82%,4.03%,4.35%,6.04%,0.97%,5%,0.24%,2.42% respectively. 5.8% of the participants were with a cross-domain degree, and 61.24% of the participants intend to pursue a doctor’s degree. There are three types of master’s degrees. 15.31% of the students are with academic degrees, 67.61% of the students are with professional degrees, and 17.08% are on-job postgraduate students.

**Table 1:** Demographic characteristics of participants (N = 1241)

*Socio demographic characteristics*	N(%)
Gender	
Male	489 (39.4%)
Female	752 (60.6%)
Academic year (%)	
First year	1028 (82.8%)
Second year	197 (15.9%)
Third year	16 (1.3%)
Professional background	
Clinical medicine	838 (67.5%)
Public health and preventive medicine	46 (3.7%)
Nursing	36 (2.9%)
Preclinical medicine	35 (2.8%)
Pharmaceutical Science	50 (4.0%)
Stormotologry	54 (4.4%)
Medical Imageology and Clinical laboratory medicine	87 (7.0%)
Clinical anesthesia	62 (5.0%)
Others	33 (2.7%)
With a Cross-Domain degree	
No	1169 (94.2%)
Yes	72 (5.8%)
Type of master degree	
Student with academic degree	190 (15.3%)
Student with professional degree	839 (67.6%)
On-job postgraduate student	212 (17.1%)
Intention to a Ph.D degree	
No	481 (38.8%)
Yes	760 (61.2%)

### Evaluation of scientific creativity and innovation ability

**Fig. 1:**
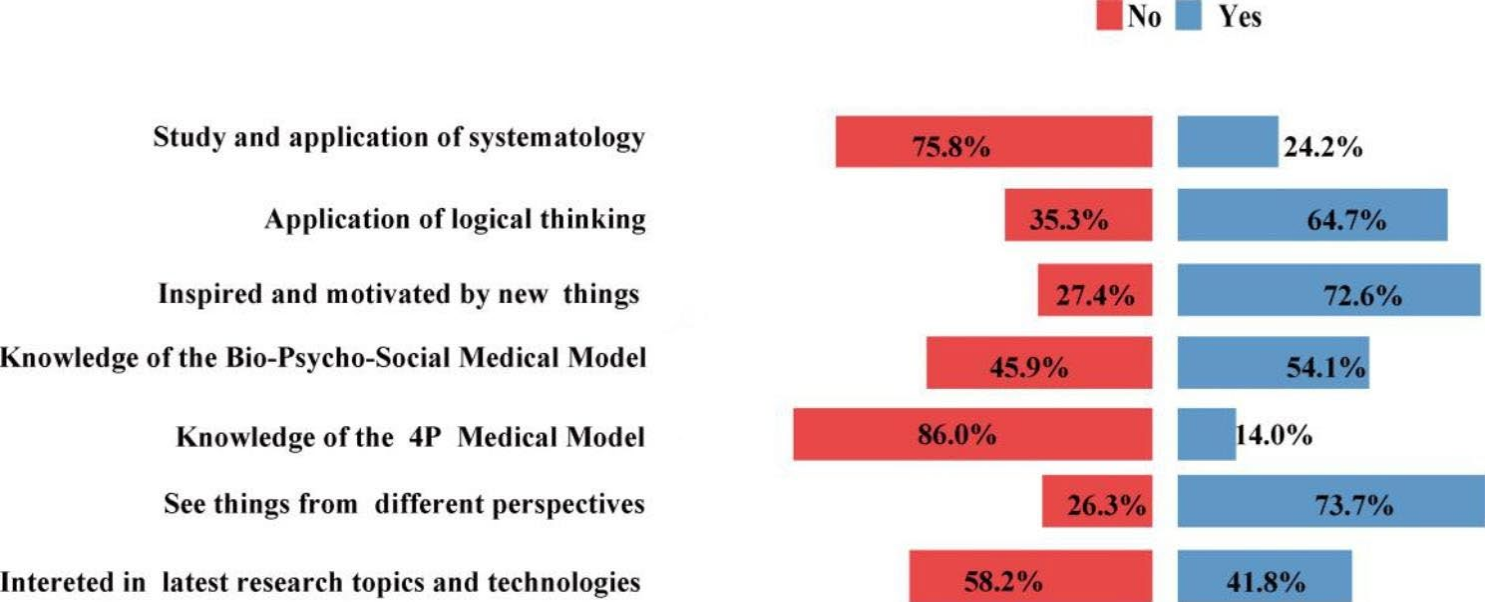
Items of creative thinking (N=1241). The distribution of proportion of the 7 items of creative thinking

**Fig. 2:**
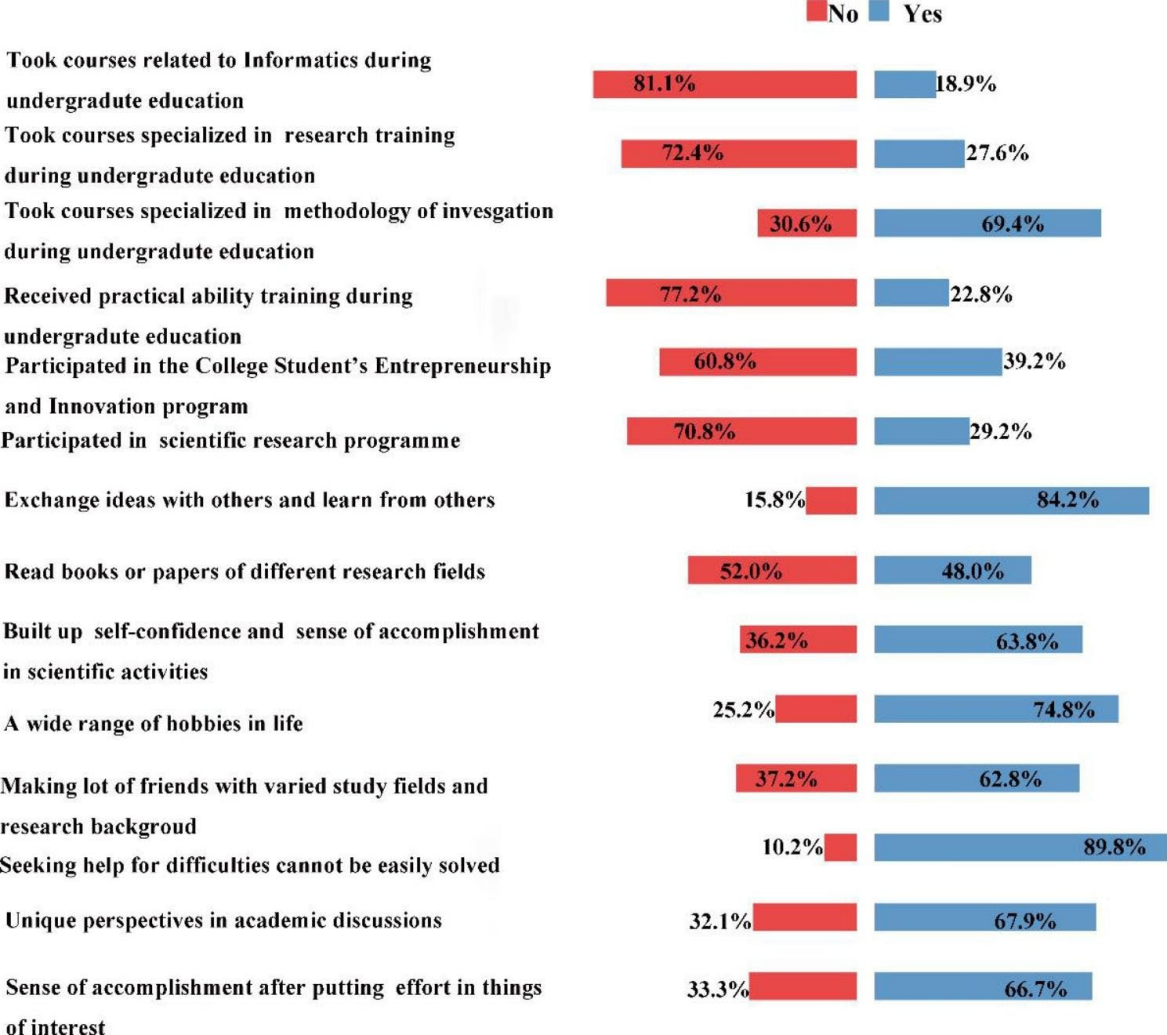
Items of cognition & skills (N=1241). The distribution of proportion of the 14 items of cognition & skills in medical science

**Fig. 3:**
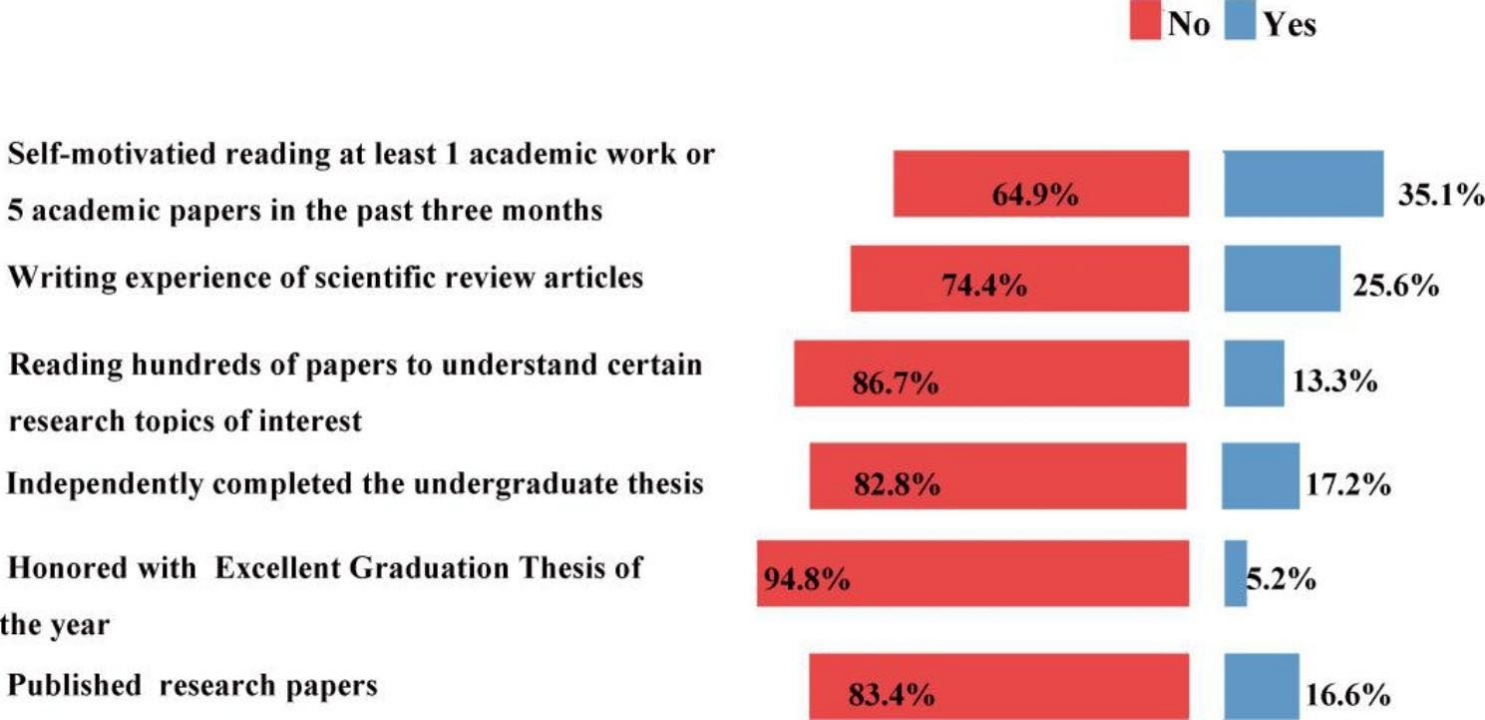
Items of academic performance (N=1241). The distribution of proportion of the 6 items of academic performance in medical science

Results of the three scales of scientific creativity and innovation ability, which consisted of creative thinking, cognition & skills in medical science, and academic performance, are shown in Figs. 1, 2 and 3. For items reflecting creative thinking as results shown in Fig. 1, only 24.17% of the participants studied and applied systematology in their study, while less than half of the students reported understanding of the Bio-Psycho-Social medical model (45.93%) and the 4P medical model (14.02%). The bright side is that the majority of them can frequently use logical thinking in the study (64.71%), or frequently inspired and motivated by something new (72.6%), or often see things from different perspectives (73.65%). Up to 69.94% of the students often pay attention to the latest topics and technologies in their research field.

Figure 2 shows the evaluation of cognition & skills in medical science, less than half of the subjects took courses related to informatics such as medical informatics (18.86%), or course specialized in scientific research training (27.56%), or received practical ability training during undergraduate education (22.8%). The only course that more than half of the students (69.38%) are familiar with is courses specialized in the methodology of investigation such as methods for social investigation or medical statistics during undergraduate education. For items of research activities, the proportion of subjects who participated in the College Student’s Entrepreneurship and Innovation program or any other scientific research programs are fairly high which are 46.82% and 29.20% respectively. Good performance was observed in terms of active learning and self-motivation with large proportions of students who often exchange ideas with others and learn from others (84.21%), or frequently read books or papers of different research fields (48.03%), or seek help when faced with difficulties that cannot be easily solved (89.77%). High level of active learning and self-motivation was also reflected by large proportions of student who often have different ideas or opinions than others when communicating with others (67.93%), or can build up good self-confidence and a sense of accomplishment in scientific activities (63.82%), or feel a sense of accomplishment after investing a lot of time and energy in something interested in (66.72%). Up to 74.78% and 62.77% of students have a wide range of hobbies in life and can make a amount of friends from varied study fields and research backgrounds.

Figure 3 displays the evaluation results of academic performance. 35.05% of student report that they were self-motivated to read at least 1 academic work or 5 academic papers in the past three months. 25.62% have experience of written scientific review articles during undergraduate or postgraduate training. 17.24% of the study subjects independently chose the research topic for their undergraduate thesis, and 5.16% were honored with Excellent Graduation Thesis for their undergraduate projects. The proportion of student that has published any research paper is 16.6%.

**Fig. 4:**
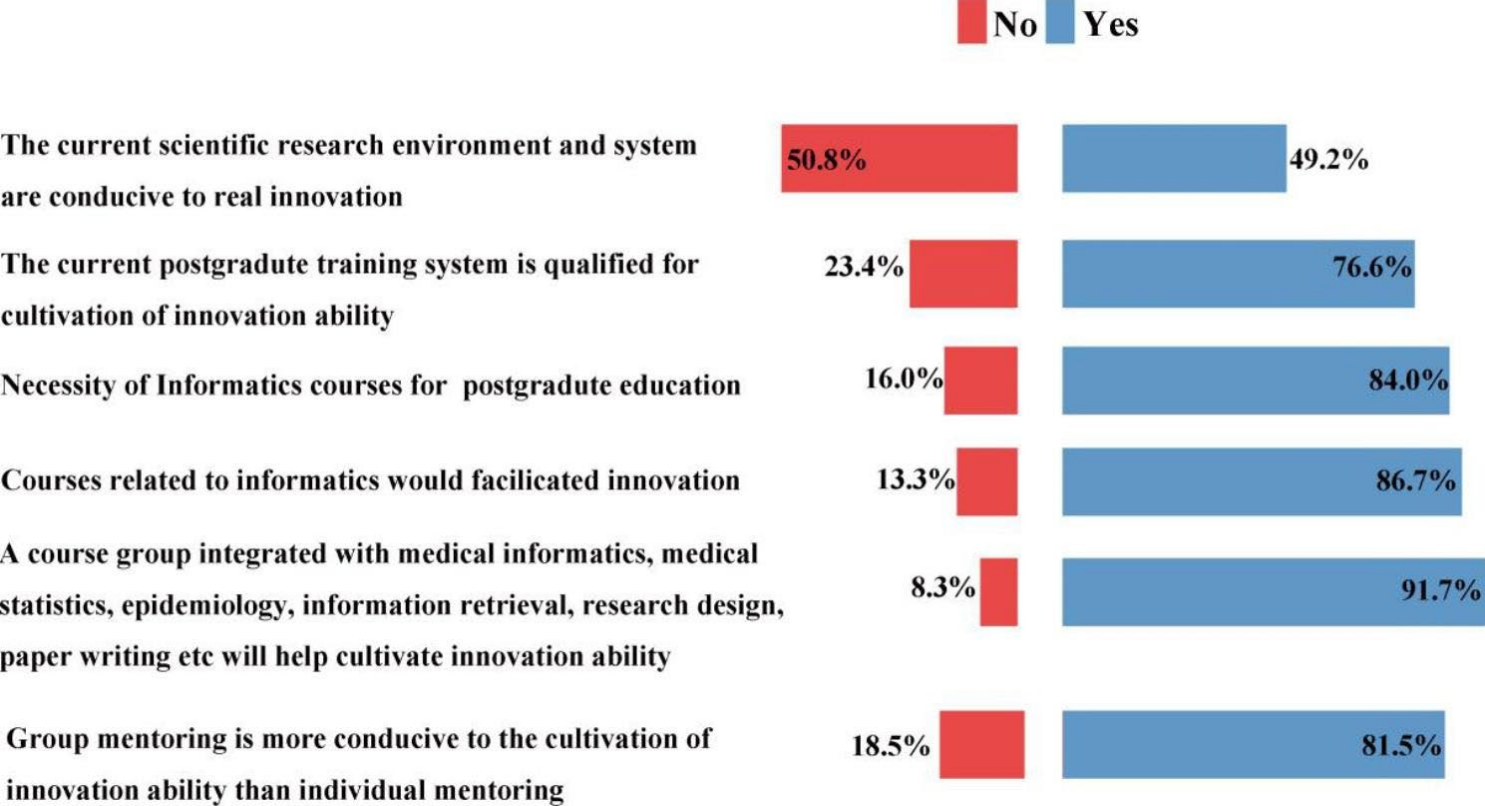
Items of attitude to the current training system (N=1241). The distribution of proportion of the 6 items of attitude to the current training system

Figure 4 displays the results of attitudes to the current training system. Half of the students (49.23%) believe that the current scientific research environment is conducive to real innovation. While most of them (76.63%) think that the current postgraduate training system is qualified for the cultivation of innovation ability. In terms of course choice, the majority think it is necessary to introduce courses in informatics such as medical informatics for postgraduate education (84.05%), and courses related to informatics would facilitate innovation (86.7%), and a course group integrated with medical informatics, medical statistics, epidemiology, information retrieval, research design, paper writing will help cultivate innovation ability (91.7%). Up to 81.55% prefer group mentoring because it is more conducive to the cultivation of innovation ability than individual mentoring.

Additionally, we investigated several details about academic activities as shown in Table 2, most of the students have a research experience of 1–2 years (67.53%), and up to 12.25% of the students have research experience of more than 5 years. Social practice, clerkship, and internship are the most important activities available for most study subjects, which was followed by the scientific program (27.2%) and field study (25.14%). Contest of scientific knowledge and skill or contest of scientific program design was reported only in 10.96% and 7.33% of the students. The most important sources of scientific data or information are public databases of scientific papers such as MEDLINE(93.88%), internet search engines such as Google(93.88%), teacher and supervisor(93.88%), followed by academic conferences(35.86% ), Wemedia (33.36%), scientific books (32.72%). Up to 81.71% of students think that it is more likely to make innovation by intercross-amalgamation of multiple disciplinary. The most important elements for innovation ability are logical thinking (69.06%), skepticism (49.56%), practical ability (46.58%), professional knowledge (44.16%), and psychological qualities (41.74%). While the most important elements of scientific paper writing are logicality (65.43%), scientific rationality (65.35%), and innovation (53.18%). The most interesting fundamental course preparing for scientific research include medical statistics (77.12%), information retrieval (72.52%), study design (70.75%), research paper writing (65.19%), and medical informatics (48.35%).

**Table 2:** Additional details about academic activities of the study subjects

Items				
Years of research experience	1-2years (67.53% )	3–4 years (20.23%)		≥ 5 years (12.25%)
What types of research and innovation activities have you participated in so far?	Yes		No	
1. Social practice	884(71.2%)		357(28.8% )	
2. Clerkship and Internship	1040(83.8%)		201(16.2%)	
3. Course design	180(14.5% )		1061(85.5% )	
4. Scientific field study	312(25.1% )		929(74.9% )	
5. Academic Reports	170(13.7%)		1071(86.3%)	
6. Scientific program	338(27.2%)		903(72.8% )	
7. Contest of scientific knowledge and skill	136(11.0%)		1105(89.0%)	
8. Contest of program design	91(7.3%)		1150(92.7%)	
For review or research work, which of the following sources do you regularly obtain data or information from?	Yes		No	
1. Database of scientific papers(MEDLINE etc.)	1165(93.9%)		76(6.1%)	
2. Internet search engine(Google, Baidu etc.)	857(69.1%)		384(30.9%)	
3. Scientific books	406(32.7%)		835(67.3%)	
4. Wemedia	414(33.4%)		827(66.6%)	
5. Academic conferences	445(35.9%)		796(64.1%)	
6. Teacher and supervisor	707(57.0%)		534(43.0%)	
7. Government websites	155(12.5%)		1086(87.5%)	
8. Annual health data released by the government	137(11.0%)		1104(89.0%)	
Which of the follow aspects do you think is more likely to make innovation for the scientific work you are working on or will work on?	Yes		No	
1. Research on specific field	386(31.1% )		855(67.6%)	
2. Multiple subject intercross-amalgamation	1014(81.7%)		227(18.3%)	
3. Emerging discipline in medical science	542(43.7% )		699(56.3%)	
4. Methodological research	388(31.3%)		853(68.7%)	
5. Theoretical research	251(20.2%)		990(79.8%)	
6. Integrated research approaches	402(32.4% )		839(67.6%)	
What qualities do you think are most important for innovation ability?	Yes		No	
1. Psychological qualities	518(41.7%)		723(58.3%)	
2. Skepticism	615(49.6%)		626(50.4%)	
3. Practical ability	578(46.6%)		663(53.4%)	
4. Logical thinking	857(69.1% )		384(30.9%)	
5. Professional knowledge	548(44.2%)		693(55.8%)	
6. Ability to generalize	275(22.2%)		966(77.8%)	
7. Team work	155(12.5%)		1086(87.5%)	
8. Communication skills	56(4.5%)		1185(95.5%)	
Which of the following should be paid more attention to in scientific paper writing?	Yes		No	
1. Integrity	580(46.7% )		661(53.3%)	
2. Logicality	812(65.4%)		429(34.6%)	
3. Scientific rationality	811(65.4%)		430(34.6%)	
4. Innovation	660(53.2%)		581(46.8%)	
5. Scientific value	602(48.5%)		639(51.5%)	
6. Authenticity	159(12.8%)		1082(87.2%)	
Which course do you think is the most important general foundation requisite to prepare you for scientific research?	Yes		No	
1. Information retrieval	900(72.5% )		341(27.5%)	
2. Medical stastitics	957(77.1%)		284(22.9%)	
3. Medical Informatics	600(48.3%)		641(51.7%)	
4. Study design	878(70.7%)		363(29.3%)	
5. Epidemiology	332(26.8%)		909(73.2%)	
6. Research paper writing	809(65.2%)		432(34.8%)	
7. Dialectics	267(21.5%)		974(78.5%)	
8. Evolution and development of health policy in China	148(11.9%)		1093(88.1%)	
9. Evidence-based medicine	439(35.4%)		802(64.6%)	
10. others	54(4.4%)		1187(95.6%)	

### Relationship between creativity and influencing factors

Table 3 presents the relationship between scientific innovation ability and its influencing factors in this study. The results of multivariate logistic correlation analysis show that there is a strong relationship between student’s creativity and factors including gender, professional background, type of master degree, and intention to a doctor’s degree. Compared with female students, male students tend to get higher scores in scientific innovation ability (*OR* = 0.69, 95%*CI*: 0.53–0.89). Compared with students of clinical medicine, students from public health and preventive medicine (*OR* = 2.91, 95%*CI*: 1.43–5.90), nursing (*OR* = 2.58, 95% *CI*: 1.20–5.55), pharmaceutical science (*OR* = 4.09, 95% *CI*: 2.06–8.13) have better performance in innovation ability. Students with professional degrees have lower scores compared with students with academic degrees (*OR* = 0.65, 95% *CI*: 0.44–0.94). While students who intend to pursue a doctor’s degree exhibited higher scientific innovation ability (*OR* = 2.15, 95% *CI*: 1.67–2.78).

**Table 3:** Results of correlation analysis of scientific innovation ability and influencing factors

Socio demographic characteristics	Univariate analysis					Multivariate analysis	
	Score of innovation ability					Score of innovation ability (High vs. Low)	
	Low (0–16)	High (17–33)	*X* ^*2*^	*p*-value		*OR*	*95%CI*
Gender			7.301	0.007			
Male	212 (43.4%)	277 (56.6%)				Ref.	
Female	385 (51.2%)	367 (48.8%)				0.69	0.53–0.89
Academic year (%)			13.790	0.001			
First year	519 (50.5%)	509 (49.5%)				Ref.	
Second year	73 (37.1%)	124 (62.9%)				1.36	0.88–2.09
Third year	5 (31.3%)	11 (68.8%)				2.24	0.73–6.87
Professional background			26.610	0.001			
Clinical medicine	418 (49.9%)	420 (50.1%)				Ref.	
Public health and preventive medicine	14 (30.4%)	32 (69.6%)				2.91	1.43–5.90
Nursing	11 (30.6%)	25 (69.4%)				2.58	1.20–5.55
Preclinical medicine	16 (45.7%)	19 (54.3%)				0.87	0.39–1.96
Pharmaceutical Science	13 (26.0%)	37 (74.0%)				4.09	2.06–8.13
Stormotologry	28 (51.9%)	26 (48.1%)				1.22	0.69–2.15
Medical Imageology and Clinical laboratory medicine	50 (57.5%)	37 (42.5%)				0.71	0.44–1.17
Clinical anesthesia	34 (54.8%)	28 (45.2%)				0.80	0.46–1.38
Others	13 (39.4%)	20 (60.6%)				1.82	0.84–3.91
With a Cross-Domain degree			3.444	0.063			
No	570 (48.8%)	599 (51.2%)				Ref.	
Yes	27 (37.5%)	45 (62.5%)				1.58	0.89–2.79
Type of master degree			24.046	< 0.001			
Student with academic degree	72 (37.9%)	118 (62.1%)				Ref.	
Student with professional degree	444 (52.9%)	395 (38.2%)				0.65	0.44–0.94
On-job postgraduate student	81 (38.2%)	131 (61.8%)				1.15	0.69–1.91
Intention to a Ph.D degree			22.425	< 0.001			
No	272 (56.5%)	209 (43.5%)				Ref.	
Yes	325 (42.8%)	435 (57.2%)				2.15	1.67–2.78

In terms of the three scales of innovation ability, the relationship between influencing factors and creative thinking, cognition & skills in medical science, and academic performance were presented in Tables 4, 5 and 6. As results are shown in Table 4, female students (*OR* = 0.58, 95% *CI*: 0.46–0.74) have lower scores in terms of creative thinking. Students in the second academic year (*OR* = 1.64, 95% *CI*: 1.09–2.48), with a cross-Domain degree (*OR* = 2.50, 95% *CI*: 1.42–4.43) and intent to a doctorate’s degree (*OR* = 1.63, 95% *CI*: 1.26–2.10) exhibit better performance in creative thinking. No sex difference was observed in terms of cognition & skills in medical science as shown in Table 5. Students from public health and preventive medicine (*OR* = 3.38, 95% *CI*: 1.64–6.99), pharmaceutical science (*OR* = 2.13, 95% *CI*: 1.14-4.00), and intent to have a doctor’s degree (*OR* = 2.20, 95% *CI*: 1.70–2.84) performed better in mastering cognition & skills in medical science. In general, female (*OR* = 0.54, 95% *CI*: 0.37–0.78), students with professional degrees (*OR* = 0.41, 95% *CI*: 0.25–0.67) are inferior to their counterparts in academic performance (Table 6). While students in the second (*OR* = 3.18, 95% *CI*: 1.91–5.31) and third academic year(*OR* = 6.95, 95% *CI*: 2.32–20.8), with background of public health and preventive medicine (*OR* = 3.74, 95%*CI*:1.64–8.52), nursing (*OR* = 5.69, 95%*CI*:2.60–12.4) and pharmaceutical science (*OR* = 4.57, 95% *CI*: 2.12–9.81), with intention to have a doctor’s degree (*OR* = 2.15, 95% *CI*: 1.45–3.19) have better academic performance.

**Table 4:** Association between creative thinking and influencing factors

Socio demographic characteristics	Univariate analysis					Multivariate analysis	
	Score of creative thinking					Score of creative thinking (High vs. Low)	
	Low (0–3)	High (4–7)	*X* ^*2*^	*p*-value		*OR*	*95%CI*
Gender			24.220	< 0.001			
Male	231 (47.2%)	258 (52.8%)				Ref.	
Female	462 (61.4%)	290 (38.6%)				0.58	0.46–0.74
Academic year (%)			22.537	< 0.001			
First year	605 (58.9%)	423 (41.1%)				Ref.	
Second year	80 (40.6%)	117 (59.4%)				1.64	1.09–2.48
Third year	8 (50.0%)	8 (50.0%)				1.25	0.43–3.62
Professional background			12.323	0.137			
Clinical medicine	448 (53.5%)	390 (46.5%)				Ref.	
Public health and preventive medicine	29 (63.0%)	17 (37.0%)				0.72	0.36–1.44
Nursing	19 (52.8%)	17 (47.2%)				0.99	0.48-2.00
Preclinical medicine	21 (60.0%)	14 (40.0%)				0.55	0.24–1.30
Pharmaceutical Science	29 (58.0%)	21 (42.0%)				1.09	0.58–2.04
Stormotologry	38 (70.4%)	16 (29.6%)				0.60	0.33–1.11
Medical Imageology and Clinical laboratory medicine	58 (66.7%)	29 (33.3%)				0.52	0.31–0.87
Clinical anesthesia	33 (53.2%)	29 (46.8%)				0.96	0.55–1.66
Others	18 (54.5%)	15 (45.5%)				1.32	0.62–2.78
With a Cross-Domain degree			4.027	0.045			
No	661 (56.5%)	508 (43.5%)				Ref.	
Yes	32 (44.4%)	40 (55.6%)				2.50	1.42–4.43
Type of master degree			24.632	< 0.001			
Student with academic degree	108 (56.8%)	82 (43.2%)				Ref.	
Student with professional degree	499 (59.5%)	340 (40.5%)				0.88	0.61–1.28
On-job postgraduate student	86 (40.6%)	126 (59.4%)				1.55	0.93–2.58
Intention to a Ph.D degree			15.358	< 0.001			
No	302 (62.8%)	179 (37.2%)				Ref.	
Yes	391 (51.4%)	369 (48.6%)				1.63	1.26–2.10

**Table 5:** Association between cognition & skills in medical science and influencing factors

Socio demographic characteristics	Univariate analysis					Multivariate analysis	
	Score of cognition & skills					Score of cognition & skills (High vs. Low)	
	Low(0–7)	High(8–14)	*X* ^*2*^	*p*-value		*OR*	*95%CI*
Gender			3.034	0.082			
Male	203 (41.5%)	286 (58.5%)				Ref.	
Female	350 (46.5%)	402 (53.5%)				0.84	0.66–1.06
Academic year (%)			0.084	0.959			
First year	460 (44.7%)	568 (55.3%)				Ref.	
Second year	86 (43.7%)	111 (56.3%)				1.04	0.68–1.57
Third year	7 (43.8%)	9 (56.3%)				1.15	0.40–3.25
Professional background			11.375	0.181			
Clinical medicine	380 (45.3%)	458 (54.7%)				Ref.	
Public health and preventive medicine	12 (26.1%)	34 (73.9%)				3.38	1.64–6.99
Nursing	14 (38.9%)	22 (61.1%)				1.76	0.85–3.65
Preclinical medicine	16 (45.7%)	19 (54.3%)				1.02	0.46–2.23
Pharmaceutical Science	18 (36.0%)	32 (64.0%)				2.13	1.14-4.00
Stormotologry	25 (46.3%)	29 (53.7%)				1.16	0.65–2.05
Medical Imageology and Clinical laboratory medicine	42 (48.3%)	45 (51.7%)				1.05	0.65–1.68
Clinical anesthesia	27 (43.5%)	35 (56.5%)				1.17	0.69–1.99
Others	19 (57.6%)	14 (42.4%)				0.83	0.39–1.75
With a Cross-Domain degree			0.995	0.318			
No	525 (44.9%)	644 (55.1%)				Ref.	
Yes	28 (38.9%)	44 (61.1%)				1.25	0.71–2.22
Type of master degree			0.050	0.975			
Student with academic degree	85 (44.7%)	105 (55.3%)				Ref.	
Student with professional degree	375 (44.7%)	464 (55.3%)				1.11	0.76–1.61
On-job postgraduate student	93 (43.9%)	119 (56.1%)				1.25	0.77–2.05
Intention to a Ph.D degree			32.541	< 0.001			
No	263 (54.7%)	218 (38.2%)				Ref.	
Yes	290 (45.3%)	470 (61.8%)				2.20	1.70–2.84

**Table 6:** Association between academic performance and influencing factors

Socio demographic characteristics	Univariate analysis					Multivariate analysis	
	Score of academic performance					Score of academic performance (High vs. Low)	
	Low (0–2)	High (3–6)	*X* ^*2*^	*p*-value		*OR*	*95%CI*
Gender			4.651	0.031			
Male	405 (82.8%)	84 (17.2%)				Ref.	
Female	656 (87.2%)	96 (13.3%)				0.54	0.37–0.78
Academic year (%)			93.894	< 0.001			
First year	924 (89.9%)	104 (10.1%)				Ref.	
Second year	128 (65.0%)	69 (35.0%)				3.18	1.91–5.31
Third year	9 (56.3%)	7 (43.8%)				6.95	2.32–20.8
Professional background			56.687	< 0.001			
Clinical medicine	734 (87.6%)	104 (12.4%)				Ref.	
Public health and preventive medicine	34 (73.9%)	12 (26.1%)				3.74	1.64–8.52
Nursing	19 (52.8%)	17 (47.2%)				5.69	2.60–12.4
Preclinical medicine	30 (85.7%)	5 (14.3%)				0.91	0.28–2.96
Pharmaceutical science	36 (72.0%)	14 (28.0%)				4.57	2.12–9.81
Stormotologry	47 (87.0%)	7 (13.0%)				1.75	0.72–4.22
Medical Imageology and Clinical laboratory medicine	78 (89.7%)	9 (10.3%)				0.65	0.29–1.43
Clinical anesthesia	59 (95.2%)	3 (4.8%)				0.22	0.06–0.79
Others	24 (72.7%)	9 (27.3%)				3.56	1.44–8.77
With a Cross-Domain degree			0.777	0.378			
No	1002 (85.7%)	167 (14.3%)				Ref.	
Yes	59 (81.9%)	13 (18.1%)				1.39	0.64–2.98
Type of master degree			83.217	< 0.001			
Student with academic degree	145 (76.3%)	45 (23.7%)				Ref.	
Student with professional degree	769 (91.7%)	70 (8.3%)				0.41	0.25–0.67
On-job postgraduate student	147 (69.3%)	65 (30.7%)				1.17	0.62–2.19
Intention to a Ph.D degree			3.173	0.075			
No	422 (87.7%)	59 (12.3%)				Ref.	
Yes	639 (84.1%)	121 (15.9%)				2.15	1.45–3.19

## Discussion

Graduate education is the main approach to training high-level innovative elites. Improving innovation ability has become a key strategic requirement for innovation-oriented national construction. At present, China’s postgraduate education has entered a stage of connotative development. With the expansion of the scale of graduate education, the problems in graduate training are also increasing. It has gradually emerged that the lack of innovation ability of graduate students is recognized as the primary problem in graduate education [14]. How to comprehensively improve the quality of postgraduate teaching has become the core task of educational reform and development. However, how to measure and evaluate scholars’ research and innovation ability is one of the unsolved problems even in the academic community, not to mention for college students at school. Data about the current cultivation and development of the innovative ability of graduated students in China is limited. So we surveyed to investigate the current situation of innovation ability cultivation of graduate students in medical school and report the potential influencing factors. To the best of our knowledge, this study was among the very limited studies to address creativity cultivation within a medical setting so far.

In our survey of creative thinking cultivation, most of the students do not have enough understanding of important basic medical philosophies for developing and reactive thinking in medical science such as systematology, Bio-Psycho-Social medical model, 4P medical model, which are basic theories and concepts for modern medical science. However, more than 70% of the students can frequently use methods of logical thinking and reasoning in their study, and reported that they can frequently inspired and motivated by something new or see things from different perspectives, or pay attention to the latest topics and technologies of the research field, which are direct indicators of creativity and innovation ability in medical science research. These results suggest that the systemic theory of medicine should be an integrated part of the curriculum system to help cultivate a holistic and systematic style of thinking for medical students [15].

Cognition & skills is an important components of creativity in medical science [16]. Results of the current study found that less than one-third of the students have received courses specialized in scientific research training or informatics courses such as medical informatics, which are critical skills for scientific research. On the other hand, students are familiar with courses specialized in the methodology of investigation such as methods for social investigation or medical statistics, because courses such as epidemiology and statistics are compulsory in undergraduate and postgraduate curriculum. Additionally, in terms of choice of courses, the majority think it is necessary to include informatics courses such as medical informatics in the medical school curriculum and courses related to informatics would facilitate innovation. And almost all the students believed that curricula integrated with medical informatics, medical statistics, epidemiology, information retrieval, research design, and paper writing will help cultivate innovation ability. In recent years, the field of biomedical research has ushered in a new era of “big data”, and students need to have a solid grasp of the basic knowledge of bioinformatics and be proficient in using bioinformatics software methods to process biomedical big data [17, 18]. Health informatics training is proposed to be included in postgraduate medical education, across all specialties in varied countries [19].

Creativity, rather than being an innate trait, can be learned. The three different components of creative behavior which include domain expertise, creative-thinking skills, and motivation, all could be influenced [20]. Good nurturing and guidance in earlier school life can stimulate creativity [21]. In the US, innovative education is promoted through an early introduction to research work and innovative thinking training [22]. So we collected data about research activities and experience at the stage of undergraduate and found that about two third of the subjects participated in the College Student’s Entrepreneurship and Innovation program or any other scientific research programs during undergraduate education, which is pretty high and probably owning to the vigorous implementation of the policy of early scientific research program in the field of higher education at the national level in China to ensure the development of scientific, technical, artistic and innovative activities of higher education institutions. Among them, the National Innovation and Entrepreneurship Training for University of PRC is the most popular and provides the feasibility for guiding undergraduate students in scientific research. It is probably one of the reasons why half of the students think that the current scientific research environment and system are conducive to innovation, and most of them think that the current graduate training system is qualified for the cultivation of innovation ability. The training mode of undergraduate scientific research team building based on scientific research projects includes literature retrieval and reading, skills training, scientific research innovation idea training, and thesis writing, etc. To a certain extent, it can make up for the learning gap of undergraduates in the field of scientific research and lay the foundation for the future training of excellent clinicians.

How to measure and evaluate scholars’ scientific research and innovation ability is one of the difficult problems that the academic community has not yet solved. Harris proposed four different evaluation indicators and methods, that is, to evaluate the scientific innovation ability of scholars through the impact, quality, importance and quantity of their research results. Papers and patents are the most important indicators to evaluate the scientific innovation ability in the current evaluation system in China. So in the survey of the evaluation of academic performance, we used research paper as one important indicator. We found that the proportion of students who have experience of written scientific review articles or has published any research paper is relatively low, which is less than half of the number of students who had experience in scientific research. According to the survey, two-thirds of the students have a research experience of 1–2 years and up to 12.25% of the students have research experience of more than 5 years. This result suggested that current the training effectiveness of early scientific research programs is worth improving. A participant learning style is associated with significantly higher academic performance. Strategies that encourage more participant-style learning in the research training may be effective in increasing academic performance among students [23].

As factors related to innovation ability, male students tend to get higher scores in creative thinking, and academic performance and performed better for scientific innovation ability, which is consistent with the results of some studies [24, 25], but not with some others [26–29]. It may be argued that gender differences in creativity may originate from cultural and social factors and some misconceptions such as “men are more intelligent and that women should make more efforts to succeed” may exaggerate gender differences. However, no difference was found in cognition & skills in medical science. Probably because female students account for the majority of postgraduate population in medical school across most medical specialties, and in general female students put more time and effort in study, so no sex difference was observed in terms of cognition & skills. We also observed differences in scientific innovation ability across medical specialties. Students from public health and preventive medicine, and pharmaceutical science have better performance in cognition & skills and academic activities, which is partly because students from those departments have more time in acquiring research skills and experience compared with students from clinical medicine or nursing [30]. And as expected, students with academic degrees and with the intention to have a doctor’s degree had higher levels of academic performance, cognition & skills, and creativity. There are mainly three types of master’s degree in China, which include academic degree, professional degrees and on-job postgraduate degrees. In general, students of academic degrees are destined for scientific research training, while students with professional degree are more focused on professional skills training in addition to scientific research training. So it’s not hard to understand that students with academic degrees and bound for a doctor’s degree performed better in cognition & skills and academic activities.

### Limitations

There are several limitations of the study. It is a cross-sectional study, and the associations found in the study must be interpreted cautiously. In addition, a specific group of students from one specific area of China was studied, a fact which makes it unwarranted to generalize the findings to other medical students from China. Further studies are suggested to verify the results reported in this study. However, the results of the study can also give a brief view of the current cultivation of medical students in medical universities at the same level in China. Other limitations are related to the questionnaire used in the study, which mainly focused on the factors from the perspective of the student themselves, and did not include external and supporting factors such as existing resources of the school, guidance from tutors, etc., which are important influence factors of student’s innovation ability. In future studies, the evaluation of innovation ability can be improved and optimized by taking more considerations on the objective aspect of supporting system in postgraduate education.

## Conclusion

Scientific research programs such as the National Innovation and Entrepreneurship Training for universities of PRC have been widely implemented in the postgraduate education system throughout the country, which plays a positive role in preparing students for their scientific research work in later study life. Most of the students are satisfied with the current scientific research environment and most of them think that the current postgraduate training system is qualified for the cultivation of innovation ability. However, the training effectiveness of the current scientific research programs is worth improving, given the fact that a large proportion of students have participated in research programs while only a small proportion of participants reported academic achievements. Multiple logistic regression results showed that among the factors studied, gender, medical specialties, and types of master degree are associated with cognition & skills, academic performance, and creativity.

The study offers practical insights for informing the development of effective strategies to enhance innovation ability in medical postgraduate students. Results of the study suggested that it will be important to incorporate more techniques of creating and improving creativity in the curricula of the current postgraduate education, especially for courses such as systemic medicine and informatics, and that guidance in the earlier school life can stimulate creativity and an early introduction into scientific research work will facilitate innovative thinking and behavior.

## Data Availability

The datasets used and analyzed during the current study are available from the corresponding author on reasonable request.

## Abbreviations

QS: questionnaire
TTCT: Torrance Test of Creative Thinking
